# Urinary sulphatoxymelatonin as a biomarker of serotonin status in biogenic amine-deficient patients

**DOI:** 10.1038/s41598-017-15063-8

**Published:** 2017-11-07

**Authors:** Marta Batllori, Marta Molero-Luis, Luisa Arrabal, Javier de las Heras, Joaquín-Alejandro Fernandez-Ramos, Luis González Gutiérrez-Solana, Salvador Ibáñez-Micó, Rosario Domingo, Jaume Campistol, Aida Ormazabal, Frederic Sedel, Thomas Opladen, Basiliki Zouvelou, Roser Pons, Angels Garcia-Cazorla, Eduardo Lopez-Laso, Rafael Artuch

**Affiliations:** 1 Clinical Biochemistry and Neuropediatric Departments, Institut de Recerca Sant Joan de Déu (IRSJD), Hospital Sant Joan de Déu, Esplugues de Llobregat, Spain; 2 Centre for Biomedical Research on Rare Diseases (CIBERER), Instituto de Salud Carlos III, Madrid, Spain; 3 Neuropediatric Department, Complejo Hospitalario Universitario de Granada, Granada, Spain; 4 Division of Pediatric Metabolism, Hospital Universitario de Cruces, Barakaldo and University of the Basque Country UPV/EHU, Vizcaya, Spain; 5 Pediatric Neurology Unit, Departament of Pediatrics, Reina Sofia University Hospital, Cordoba, Spain; 6 Neuropediatric Department, Hospital Infantil Universitario Niño Jesús, Madrid, Spain; 7 Neuropediatric Department, Hospital Clínico Universitario Virgen de la Arrixaca, Murcia, Spain; 8 MedDay Pharmaceuticals, ICM-Brain and Spine Institute-IPEPs, Groupe Hospitalier Pitié Salpêtrière, 47 Boulevard de l′Hopital, 75013 Paris, France; 9 Department of Child Neurology and Metabolic Disorders, University Children’s Hospital, Heidelberg, Germany; 10 Agia Sofia Hospital, Special Pediatric Neurology Unit, First Department of Pediatrics, National and Kapodistrian University of Athens, Athens, Greece; 11 Pediatric Neurology Unit, Department of Pediatrics, Reina Sofia University Hospital, Maimonides Biomedical Research Institute of Cordoba (IMIBIC), Cordoba, Spain

## Abstract

Melatonin is synthesized from serotonin and it is excreted as sulphatoxymelatonin in urine. We aim to evaluate urinary sulphatoxymelatonin as a biomarker of brain serotonin status in a cohort of patients with mutations in genes related to serotonin biosynthesis. We analized urinary sulphatoxymelatonin from 65 healthy subjects and from 28 patients with genetic defects. A total of 18 patients were studied: 14 with autosomal dominant and recessive guanosine triphosphate cyclohydrolase-I deficiency; 3 with sepiapterin reductase deficiency; and 1 with aromatic L-amino acid decarboxylase deficiency. Further 11 patients were studied after receiving serotoninergic treatment (serotonin precursors, monoamine oxidase inhibitors, selective serotonin re-uptake inhibitors): 5 with aromatic L-amino acid decarboxylase deficiency; 1 with sepiapterin reductase deficiency; 3 with dihydropteridine reductase deficiency; and 2 with 6-pyruvoyltetrahydropterin synthase deficiency. Among the patients without therapy, 6 presented low urinary sulphatoxymelatonin values, while most of the patients with guanosine triphosphate cyclohydrolase-I deficiency showed normal values. 5 of 11 patients under treatment presented low urine sulphatoxymelatonin values. Thus, decreased excretion of sulphatoxymelatonin is frequently observed in cases with severe genetic disorders affecting serotonin biosynthesis. In conclusion, sulphatoxymelatonin can be a good biomarker to estimate serotonin status in the brain, especially for treatment monitoring purposes.

## Introduction

**Figure 1:**
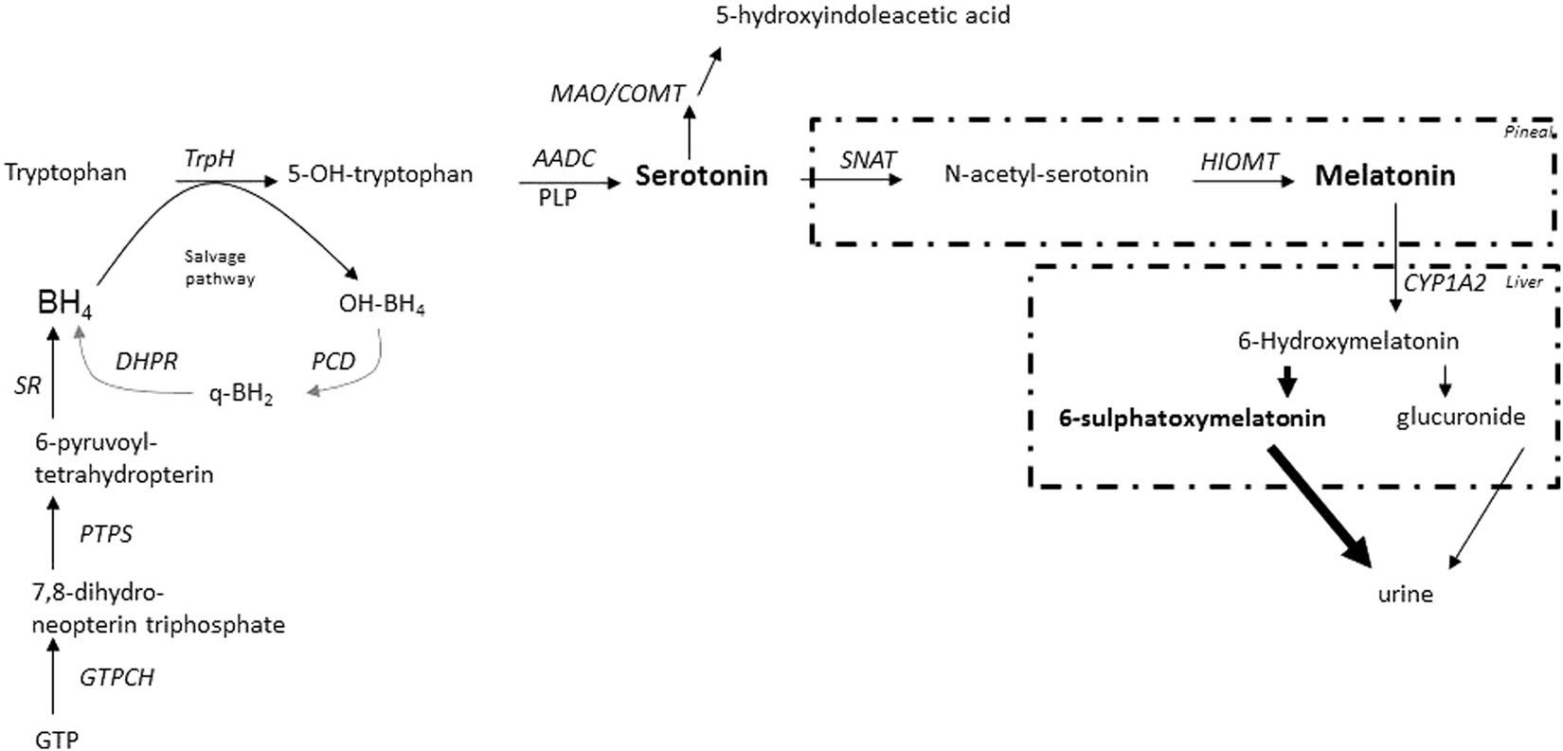
Melatonin pathway. Melatonin synthesis occurs in pineal gland (and other tissues) and its metabolism produces 6-sulphatoxymelatonin, the main urine melatonin metabolite. Serotonin and melatonin are marked in bold type. Synthesis and salvage pathways (grey arrows) of the tetrahydrobiopterin (BH_4_) are also represented. Enzymes are in cursive. AADC: aromatic L-amino acid decarboxylase; BH_4_: tetrahydrobiopterin; COMT: catechol O-methyltransferase; CYP1A2: cytochrome P450 isoform CYP1A2; DHPR: dihydropteridine reductase; GTP: guanosine triphosphate; GTPCH: GTP cyclohydrolase I; HIOMT: 5-hydroxyindole-O-methyltransferase; MAO: monoamine oxidase; OH-BH_4_: hydroxy-tetrahydrobiopterin; PCD: pterin-4a-carbinolamine dehydratase; PTPS: 6-pyruvoyl-tetrahydropterin synthase; q-BH_2_: quinonoid-dihydrobiopterin; SNAT: serotonin N-acetyltransferase; SR: sepiapterin reductase; TrpH: tryptophan hydroxylase; PLP: pyridoxal phosphate.

Melatonin (5-methoxy-N-acethyltriptamine) is secreted by the pineal gland and is synthesized from serotonin. Melatonin synthesis is regulated by two specific enzymes: serotonin-N-acetyl transferase (SNAT, EC 2.3.1.5), which is a rate-limiting enzyme, and 5-hydroxyindole-O-methyl transferase (HIOMT EC 2.1.1.4), which transfers a methyl group from S-adenosylmethionine to 2-hydroxyl of N-acetylserotonin (Fig. 1). Melatonin is released from the pineal gland and enters the circulation. Other melatonin sources are the retina, gut, skin, platelets and bone marrow, but their contribution to circulating melatonin is less relevant than that of pineal gland^1^. Melatonin is metabolized in the liver to 6-hydroxymelatonin by cytochrome CYP1A2 (EC 1.14.14.1), and it is excreted in urine as sulphatoxymelatonin (aMT6s) and, to a lower extent, as glucuronide conjugate^1^. Urine aMT6s excretion closely correlates to the plasma melatonin profile^1,2^ and is a good indicator of melatonin secretion from the pineal gland^3^. Thus, it has been suggested that the measurement of urinary aMT6s may be a good biomarker of serotonin status in the brain^4^. Yano *et al*. reported that blood melatonin and urine aMT6s levels may serve as biomarkers reflecting brain serotonin synthesis in subjects with phenylketonuria (PKU)^4^.

There are several genetic alterations affecting brain serotonin and dopamine biosynthesis (Fig. 1): aromatic L-amino acid decarboxylase (AADC) deficiency (OMIM#608643), pyridoxal phosphate (PLP) deficiency (pyridoxamine 5-phosphate oxidase deficiency, OMIM#610090), antiquitin deficiency (OMIM#266100) and tetrahydrobiopterin (BH_4_) disorders including the following deficiencies: dominant and recessive form of guanosine triphosphate cyclohydrolase-I (GTPCH-1, OMIM#128230 and OMIM#233910 respectively), 6-pyruvoyltetrahydropterin synthase (PTPS, OMIM#261640), sepiapterin reductase (SR, OMIM#612716), dihydropteridine reductase (DHPR, OMIM#261630) and primapterinuria (OMIM#264070). Other disorders such as tyrosine hydroxylase deficiency and the recently reported transportopathies^5,6^ impair dopamine biosynthesis in particular^7^. The clinical presentation of these disorders include symptoms related to autonomic dysfunction, which manifest as sweating, temperature dysregulation, hypersalivation, nasal congestion and psychiatric signs^8^ such as bad behaviour and autistic features. Movement disorders are caused mainly by dopamine dysregulation, which includes gait disturbances, dystonia, dyskinesia, Parkinsonism, tremor, oculogyric crises, palpebral ptosis and axial hypotonia^7,9^. However, the symptoms that are thought to be related to serotonin deficiency are more difficult to clinically assess.

To estimate serotonin deficiency in these conditions, the analysis of cerebrospinal fluid (CSF) 5-hydroxyindoleacetic acid (5HIAA) is the most appropriate tool^10^. Furthermore, most of the patients harbouring these disorders received different treatment approaches to restore normal serotonin (and dopamine) levels in the brain. To evaluate the biochemical response to treatment, patients often undergo a lumbar puncture for the monitoring of brain serotonin status by 5HIAA quantification.

To evaluate whether urine aMT6s is a reliable biomarker for serotonin status in the brain, we first established normal values for aMT6s in urine in a healthy population. In a second step, we analysed urine samples from patients with pathogenic mutations in genes involved in the serotonin biosynthetic pathway.

## Methods

### Subjects

The reference values (RVs) for urinary aMT6s were established in 65 healthy control subjects (36 females; age range 2–61 years; mean = 10.0) with similar age to our patient population. Lighting environment during the night when the morning urine was recovered was controlled (in darkness). The duration of sleep on that night was between 8–11 hours. Exclusion criteria were the presence of any pharmacological conditions affecting serotonin status, evidence of drug or alcohol abuse and a history of shift work or travel across two or more time zones in the preceding month. After establishing the RVs, urine aMT6s was studied in 28 patients with genetic defects affecting serotonin biosynthesis (age range 2–55 years; mean = 13.0). Details of these patients are described in Tables 1 and 2. Out of these 28 cases, patients 1–18 were patients without treatment (naive patients) or patients on L-dopa/carbidopa therapy (Table 1), and the rest of cases (18bis-28) were under serotoninergic treatment (Table 2) including serotonin precursors (5-hydroxytryptophan, (5HTP)), monoamine oxidase inhibitors (MAOIs), selective serotonin re-uptake inhibitors (SSRI), cofactors that enhance serotonin/melatonin biosynthesis (BH_4_, PLP and folinic acid) and melatonin treatment. Other treatments that were applied are also stated in Tables 1 and 2. Patient 18 was studied twice, at diagnosis (stated as patient 18 in Table 1) and after 3 months on MAOIs therapy (stated as patient 18bis in Table 2

**Table 1:** Biochemical, genetic and medical information of 18 patients (naive and under L-dopa/carbidopa treatment) (patients 1–18).

Patients	Date of birth (age at analyses time)	Disease (#OMIM) *Gene* Mutations	aMT6s µmol/mol creatinine (ref. values)	% aMT6s reduction	CSF 5HIAA nmol/L (reduction, %)	Treatment	Dose (units)	Duration
1	1960 (56 years)	dGTPCH (#233910)*GCH1*c.265C>T (p.Q89X)	4.1(6.3–37.9)	−34.9	NP	L-dopa/carbidopa	300 mg/d	15y
2	1963 (53 years)	dGTPCH (#233910)*GCH1*c.265C>T (p.Q89X)	8.4(6.3–37.9)	No reduction	NP	L-dopa/carbidopa	400 mg/d	5y
3	1966 (50 years)	dGTPCH (#233910)*GCH1*c.265C>T (p.Q89X)	18.2(6.3–37.9)	No reduction	NP	L-dopa/carbidopa	375 mg/d	25y
4	1996 (20 years)	dGTPCH (#233910)*GCH1*c.265C>T (p.Q89X)	12.7(6.3–37.9)	No reduction	NP	L-dopa/carbidopa	75 mg/d	15y
5	2002 (14 years)	dGTPCH (#233910)*GCH1*c.265C>T (p.Q89X)	19.8(11.9–66.2)	No reduction	236 (no reduction) At 1 y of age (diagnose time)	L-dopa/carbidopa	130 mg/d	12y
6	1992 (24 years)	dGTPCH (#233910)*GCH1*c.265C>T (p.Q89X)	38(6.3–37.9)	No reduction	NP	No. Naive patient		
7	1998 (18 years)	dGTPCH (#233910)*GCH1*c.235_240delCTGAGC (p.L79_S80del)	21.2(6.3–37.9)	No reduction	NP	L-dopa/carbidopa	62.5 mg/d	10 y
						Trihexypenidyl	6 mg/d	6y
8	1963 (52 years)	dGTPCH (#233910)*GCH1*c.235_240delCTGAGC (p.L79_S80del)	6.5(6.3–37.9)	No reduction	NP	No. Naive patient		
9	2006 (10 years)	dGTPCH (#233910)*GCH1*c.265C>T (p.Q89X)	13.4(11.9–66.2)	No reduction	NP	No. Naive patient		
10	1974 (42 years)	dGTPCH (#233910)*GCH1*c.265C>T (p.Q89X)	12.1(6.3–37.9)	No reduction	NP	L-dopa/carbidopa	200 mg/d	7 y
11	1950 (66 years)	dGTPCH (#233910)*GCH1*c.265C>T (p.Q89X)	18.1(6.3–37.9)	No reduction	NP	L-dopa/carbidopa	NA	NA
12	1971 (45 years)	dGTPCH (#233910)GCH1c.68C>T (p.P23L)	19.8(6.3–37.9)	No reduction	NP	No. Naive patient		
13	1967 (49 years)	dGTPCH (#233910)GCH1c.265C>T (p.Q89X)	1.9(6.3–37.9)	−69.8	NP	No. Naive patient		
14	2006 (10 years)	rGTPCH (#128230)GCH1c.68C>T/c.265C>T(p.P23L/p.Q89X)	34.2(11.9–66.2)	No reduction	NP	L-dopa/carbidopa	120 mg/d	4y
15	1997 (19 years)	SR (#612716)*SPR*c.304G>T/c.448A>G(p.G102C/p.R150G)	2.5(6.3–37.9)	–60.3	25 (−60.3) At 11 y of age (diagnose time)	L-dopa/carbidopa	180 mg/d	8 y
16	1992 (24 years)	SR (#612716)*SPR*c.304G>T/c.448A>G(p.G102C/p.R150G)	4.8(6.3–37.9)	−23.8	NP	L-dopa/carbidopa	300 mg/d	7 y
17	1990 (26 years)	SR (#612716)*SPR*c.304G>T/c.448A>G(p.G102C/p.R150G)	0.8(6.3–37.9)	−87.3	NP	No. Naive patient		
18*	2014 (1 years)	AADC (#608643)*DDC*c.1041+1G>C/c.323G>A(IVS11 ds G-C +1 /p.S108N)	1.1(19.4–79.1)	−94.3	1 (−99.4) At 1 y of age (diagnose time)	No. Naive patient		

*Patient 18 was studied in baseline conditions. The percentage of aMT6s reduction represents the decrease in aMT6s levels compared with the lower limit of the reference values in each age group. mo: months; ref. values: reference values; w- weeks; y: years; NA: not available; NP: not performed.

**Table 2:** Biochemical, genetic and medical information of 11 patients on treatment related to serotoninergic pathway (patients 18bis–28).

Patients	Year of birth (age at analyses time)	Disease (#OMIM) *Gene* Mutations	aMT6s µmol/mol creatinine (ref. values)	% aMT6s reduction	CSF 5HIAA nmol/L (reduction, %)	Treatment	Dose (units)	Duration
18 bis*	2014 (2 years)	AADC (#608643)*DDC*c.1041+1G>C/c.323G>A(IVS11 ds G-C +1 /p.S108N)	9.2(19.4–79.1)	−52.6	**No further analyses on treatment**	**Selegiline**	0.5–0.5–0 mg	3 mo
19	2006 (10 years)	AADC (#608643)*DDC*c.1040G>A/c.1040G>A(p.R347Q/p.R347Q)	3.5(11.9–66.2)	−70.6	10 (−94.1) At 1.5 y of age(diagnose time)	PLP	100 mg/d	3 mo
						**Selegiline**	10mg/d	2 w
						Ropinirol	5 mg /d	2 w
20	2009 (6 years)	AADC (#608643)*DDC*c.1040G>A/c.1040G>A(p.R347Q/p.R347Q)	7.4(19.4–79.1)	−61.8	63 (−62.9) At 6 mo of age (diagnose time)	PLP	100 mg /d	2 w
						**Selegiline**	10 mg /d	2 w
21	2013 (3 years)	AADC (#608643)*DDC*c.799T>C /c.799T>C(p.W267R/p.W267T)	20.7(19.4–79.1)	No reduction	27 (−84.1) At 1 y of age(diagnose time)	**Selegiline**	10 mg/d	Chronic treatment
						Ropinirol	2,25 mg/d	
22	2008 (8 years)	AADC (#608643)*DDC*c.367G>A/c.734C>T(p.G123R)/p.T245I)	285(11.9–66.2)	Increase	25 (−85.3) At 11 mo of age (diagnose time)	Folinic acid	15 mg/d	6 y
						PLP	100–100–100 mg	6 y
						Bromocriptine	7.5–7.5–7.5 mg	7 y
						**Selegiline**	10.5–10.5–10.5 mg	7 y
						**Fluoxetine**	6 mg/d	4 y
						**Melatonin**	3 mg (12 drops) at night	5 y
23	2004 (11 years)	SR (#612716)*SPR*c.751A>T/c.751A>T (p.K251X/p.K251X)	1.1(11.9–66.2)	−90.7	9 (−94.7) At 23 mo of age (diagnose time)	L-dopa/carbidopa	120 mg/d	10 y
						**5HTP**	60 mg/d	10 y
						Folinic acid	20 mg/d	10 y
24	2011 (5 years)	DHPR (#261630)*QDPR*c.661C>T/c.609dupA (p.R221Ter/P204TfsTer7)	900(19.4–79.1)	Increase	158 (no reduction) At 12 mo of age (analysis on treatment)	L-dopa/carbidopa	160 mg/d	Chronic treatment
						**5HTP**	50 mg/d	
						Folinic acid	20 mg/d	
						BH_4_	300 mg/d	
						Clonazepam	1,5 mg/d	
						Zonisamide	100 mg/d	
						Prednisone	8 mg q.o.d	
						**Fluoxetine**	5 mg/d	
25	2011 (5 years)	DHPR (#261630)*QDPR*c.609dupA/c.466G>A (p.P204TfsTer7)/p.A156T)	19.5(19.4–79.1)	No reduction	9 (−94.7) At 2 y of age (diagnose time)	L-dopa/carbidopa	100 mg/d	Chronic treatment
						**5HTP**	50 mg/d	
26	2011 (5 years)	DHPR (#261630)*QDPR*c.384_387del/c.665C>T (p.Gly129Alafs*/p.Pro222Leu)	29.5(19.4–79.1)	No reduction	133 (no reduction) At 5 years of age (analysis on treatment)	L-dopa/carbidopa	13.5 mg/d	Chronic treatment
						**5HTP**	8.5 mg/d	
27	2005 (11 years)	PTPS (#261640)*PTS*c.98A>G / c.297C>A(p.Asp33Gly/p.Tyr99Ter)	19.6(11.9–66.2)	No reduction	65 (−61.8) At 1 year of age (on treatment) 169 (no reduction)At 11 years of age(on treatment)	L-dopa/carbidopa	33 mg/6 h	Chronic treatment
						**5HTP**	36 mg/6h	
						BH_4_	150 mg/d	
						Calcium folinat	15 mg/d	
						Levothyroxine	50 µg/d	
28	2005 (11 years)	PTPS (#261640)*PTS*c.260C>T/c.260C>T(p.P87L/p.P87L)	2.2(11.9–66.2)	−81.5	194 (no reduction) At 8 years of age(analysis on treatment)	L-dopa/carbidopa	400 mg/d	Chronic treatment
						**5HTP**	100 mg/d	
						BH_4_	100 mg/d	

*Patient 18bis was studied after serotoninergic treatment. Serotoninergic drugs are highlighted in bold. The percentages of aMT6s and cerebrospinal fluid (CSF) 5HIAA reduction were calculated from the lower limit of the reference values for each age group. aMT6s: urine 6-sulphatoxymelatonin expressed as µmol 6-sulphatoxymelatonin/mol creatinine. CSF 5HIAA values are expressed as nmol/L. q.o.d.: every other day. 5HTP: 5-hydroxytryptophan; BZP: benzodiazepines; Dopamine ag: dopamine agonist; MAOIs: monoamine oxidase inhibitors; mo: months; PLP: pyridoxal phosphate; ref. values: reference values; w- weeks; y: years.

).

### Ethical issues

All samples from the patients were obtained in accordance with the 2013 revised Helsinki Declaration of 1964. For biochemical and genetic investigations, informed consent was collected from patients or their guardians. The Ethical Committee of Sant Joan de Déu Hospital approved the study.

### Samples

For both the controls and the patients, the first morning urine samples were collected, centrifuged and stored at −20 °C until analysis. From the 28 patients, a total of 29 urine samples were collected, because, in one case with AADC deficiency, we analysed one urine sample at baseline conditions and again after therapy (patient 18). A total of 18 urine samples were taken from patients not treated with serotoninergic therapy (Table 1, patients 1–18). A further 11 urine samples were collected from subjects on different combinations of serotoninergic drugs (Table 2, patients 18bis-28).

In Tables 1 and 2, we also stated CSF 5HIAA values when available (13 out of 28 patients). Most of the CSF analyses were done during the treatment monitoring process (Table 2).

### Urinary aMT6s analysis validation

#### Assay variables

**Figure 2:**
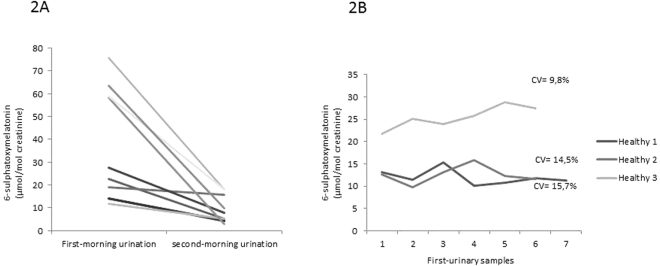
Assay variables. (**A**) Urine 6-sulphatoxymelatonin (aMT6s) values in the first and second morning urine samples in 10 healthy subjects. (**B**) Intra-individual variation of urinary aMT6s excretion in three healthy volunteers during 7 consecutive days.
- To study the intra-individual variations of urinary aMT6s excretion, urinary aMT6s was analysed in three healthy volunteers during 7 consecutive days.
- To evaluate assay imprecision, we calculated the within-run and between-run coefficients of variation (CV = standard deviation/mean aMT6s values*100) in 10 replicates.
- After these metrological studies, RVs were established in the first morning urine samples, as reported by other groups^4,11,12^.

- To study urinary melatonin excretion changes related to the time of sampling, we analysed aMT6s values in the first and second morning urine samples in 10 healthy subjects (age range: 4–33 years) (Fig. 2A).

Urinary aMT6s was analysed by duplicate using a competitive ELISA kit (IBL; ref RE54021) and the optical density measured with a photometer (ATOM S.A. Barcelona, Spain) at 405 nm and 630 nm. Coefficients of variation of the duplicates were calculated. Creatinine concentration was determined by an automated spectrophotometric assay in the Architect c8000 analyser (Abbott). Results are expressed as µmol aMT6s/mol creatinine.

#### Statistical analysis

The Kolmogorov–Smirnow test was used to assess the distribution of the data. Because the data did not follow a Gaussian distribution, different non-parametric tests were applied. The Spearman simple correlation test was used to determine the correlations between urinary aMT6s and patient age. The Kruskal–Wallis and Mann–Whitney U tests were used to compare urinary aMT6s excretion among the different age groups and also between GTPCH naive patients and those under L-dopa/carbidopa treatment. Statistical calculations were performed using SPSS 23.0 software.

### Data availability

The datasets generated during and/or analysed during the current study are available from the corresponding author on reasonable request.

## Results

### Assay variables

The first morning urine sample showed the highest aMT6s concentrations in the 10 healthy cases, which remarkably dropped down in the second morning sample (Fig. 2A). The mean reduction from the first to the second urine samples was 68.7%.

The intra-individual variation studied in the three volunteers showed a CV value of 14.5%, 15.1% and 9.9%, respectively (Fig. 2B).

Regarding the imprecision assay studies, within-run CV was 17.0% for aMT6s = 11.5 ng/mL and between-run CV was 10.6% for aMT6s = 114.6 ng/mL.

Urinary aMT6s was analysed by duplicate. In the 80% studied samples, the variation of the duplicates was <10%, in the 10% studied samples was 10–15% and in the other 10% of studied samples the variation was >15%.

### Establishment of reference values

**Figure 3:**
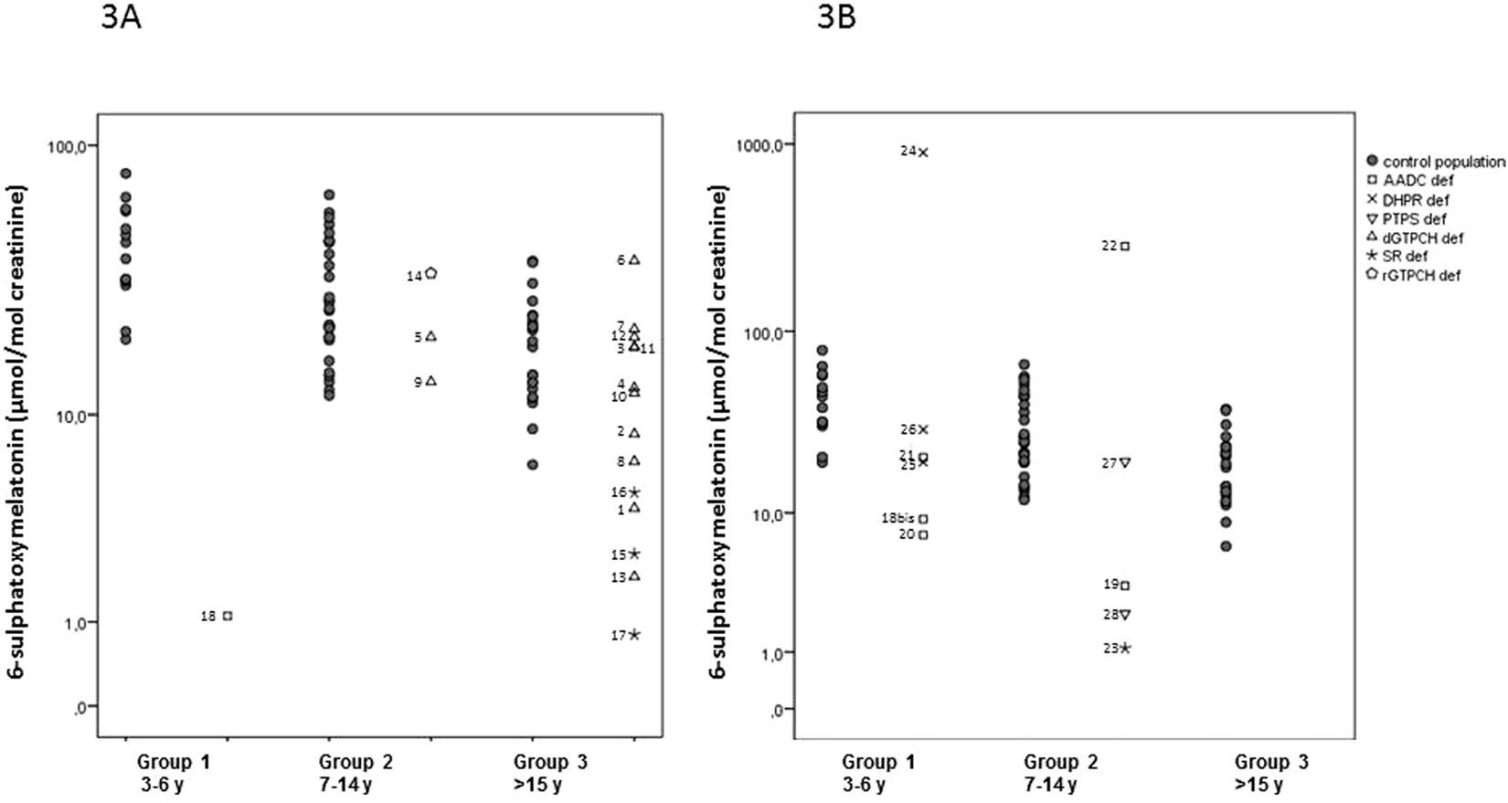
Representation of urine 6-sulphatoxymelatonin (aMT6s) values from the reference population and patients with serotonin defects without serotoninergic treatment (**A**) and with serotoninergic treatment (**B**). Each disease is represented by different geometric shapes: square-AADC deficiency; cross-DHPR deficiency; inverted triangle-PTPS deficiency; triangle-adGTPCH deficiency; star-SR deficiency, and hexagon-arGTPCH deficiency. Each number matches with the patient’s number as shown in Tables 1 and 2. AADC: aromatic L-amino acid decarboxylase; DHPR: dihydropteridine reductase; GTPCH: guanosine triphosphate cyclohydrolase I; PTPS: 6-pyruvoyl-tetrahydropterin synthase; SR: sepiapterin reductase.

In the healthy population (n = 65), a negative correlation was observed between urine aMT6s in the first morning urine sample and the age (r = −0.591; p < 0.001, Spearman test). RVs for our population were stratified according to age in three different groups as statistical differences were found (U = 28.8, p < 0.001 in groups 1–2; U = 9.0, p < 0.001 in groups 1–3; U = 34.0, p < 0.001 in groups 2–3; Table 3; Fig. 3).

**Table 3:** Urine 6-sulpatoxymelatonin (aMT6s) values of control population.

**Group**	**Median (µmol aMT6s /mol creatinine)**	**Min-Max**
1 (2–6 years) n = 14	41.5	19.4–79.1
2 (7–14 years) n = 29	25.3	11.9–66.2
3 ( > 15 years) n = 22	18.6	6.3–37.9

Reference values were established in three different groups and are expressed as median, minimum and maximum values. The Mann-Whitney U test showed statistical differences between groups 1 and 2 (U = 28.8; p < 0.001), between groups 1, 2 and 3 (U = 9.0; p < 0.001) and between groups 2 and 3 (U = 34.0; p < 0.001). Units = µmol aMT6s /mol creatinine.

### Patients not treated with serotoninergic treatment (Table 1)

Naive patients (patients 6, 8, 9, 12, 13, 17 and 18) and cases under L-dopa/carbidopa therapy (patients 1–5, 7, 10, 11, 14–16) were included (n = 18). Out of 18 cases, we studied 13 patients with autosomal dominant GTPCH (adGTPCH) deficiency (patients 1–13); 1 patient with autosomal recessive GTPCH (arGTPCH) deficiency (patient 14); 3 patients with SR deficiency (patients 15–17); and 1 patient with AADC deficiency (patient 18). We compared the results with those from our RVs (Table 1 and Fig. 3A). Regarding patients with adGTPCH deficiency (n = 13), only 2 presented decreased urine aMT6s values (patients 1 and 13; 34.9% and 69.8% of aMT6s reduction, respectively). No statistical differences were observed between adGTPCH patients and the control group. Patient with arGTPCH form (patient 14) had normal aMT6s values, whereas patients with SR and AADC deficiencies had very low urine aMT6s values compared to their age reference group.

No differences were observed in urinary aMT6s values when compared GTPCH naive patients (median = 13.4) and those under L-dopa/carbidopa therapy (median = 18.1; U = 20.5; p = 0.797).

### Patients under serotoninergic treatment (Table 2)

We studied five patients with AADC deficiency (patients 18bis–22), one with SR deficiency (patient 23), three with DHPR deficiency (patients 24–26) and two with PTPS deficiency (patients 27–28). Details of the different treatments and results are stated in Table 2 and Fig. 3B. Three AADC deficiency cases (patients 18bis–20) presented low urine aMT6s values, while patient 21 showed normal aMT6 values. Patient 18bis was studied twice, at diagnosis (stated as patient 18) and after 3 months on MAOIs therapy (stated as patient 18bis). In this condition, aMT6s values increased from 1.1 to 9.2 µmol/mol creatinine, although they remained below the RVs (Table 1 and Table 2). Patient 22 was on melatonin therapy and therefore had a higher aMT6s concentration than RVs.

Regarding patients with pterin-related defects, some showed decreased aMT6s excretion despite receiving 5HTP treatment. Among them, the SR-deficient case (patient 23) and PTPS-deficient case (patient 28) showed remarkably reduced urinary aMT6s excretion (90.7% and 81.5%, respectively), while the DHPR-deficient cases (patients 24–26) and one PTPS-deficient case (patient 27) presented normal aMT6s values.

## Discussion

This is the first report that assessed urinary aMT6s concentrations in genetic conditions that cause a severe effect on brain serotonin synthesis. Regarding assay variables, we corroborated that the first morning urine was the most suitable sample because the majority of melatonin is metabolized and excreted into urine as aMT6s^4^. Thus, any urine leak during the night would noticeably decrease aMT6s concentrations^12^. This fact would explain the unexpected low aMT6 value observed in two of our patients under serotoninergic treatment, who probably had troubles in urine sample collection (patient 18, who was 2 years old, and patient 23, because he had urinary continence problems).

Our analytical studies have shown that intra-individual variation only ranged from 9.9% to 15.1% (Fig. 2B). Furthermore, the metrological variations obtained were also reasonable for a proper interpretation of the results. Only 10% of patients presented CV of the duplicates higher than 15%, which was probably due to cross-reaction phenomena related to immune-based laboratory methods^11^. Thus, such samples were not considered in the study and should be reanalysed.

Regarding RVs, we established three different age groups (Table 3). Our results can be explained by the fact that melatonin synthesis reaches the highest rate at the age of 3–6 years, and later it decreases progressively by 80% until adult age^1^. These RVs are similar to others previously reported^13^, which supports the reproducibility of the ELISA method for aMT6s quantification in different populations.

Considering patients without serotoninergic treatment we studied 13 patients with adGTPCH deficiency, and 11 of them presented normal values of aMT6s. The adGTPCH deficiency is the mildest disease among those studied here, because the patients have a mild to moderate reduction in dopamine and serotonin biomarkers in CSF and were even normal in some cases. In fact, in patient 5 (the index case of one family^14^), CSF 5HIAA concentrations were normal at the time of diagnosis, which suggests minimal or no alteration in brain serotonin status. Moreover, most of our adGTPCH-deficient patients harboured the same mild mutation (p.Q89X) at the *GCH1* gene as patient 5, and this fact would explain that adult cases from this cohort present a very mild (or even symptom-free) phenotype, as previously reported.

arGTPCH deficiency usually show PKU and has an early onset with a more severe clinical course than the adGTPCH deficiency^15^. Urine aMT6s levels were also normal in one case (patient 14) with arGTPCH deficiency, who showed normal phenylalanine levels and a phenotype resembling the dominant form of GTPCH deficiency, which suggested high GTPCH residual activity.

SR deficiency is inherited in an autosomal recessive manner. Patients present with a diurnally fluctuating motor disorder, and in most cases, it is associated with cognitive delay and severe neurologic dysfunction. The three patients reported here are siblings and they showed an important reduction of aMT6s levels (60.3%, 23.8% and 87.3%). In the index case (patient 15), the reduction of CSF 5HIAA at the time of diagnosis was also remarkable (Table 1). These three patients presented a mild phenotype with a late-onset presentation^16^. Moreover, they were under treatment with only L-dopa/carbidopa, as 5HTP was trialled some years ago, but the treatment was discontinued due to side effects (vomiting and diarrhoea). They presented a novel mutation in the *SPR* gene that affects splicing, which was reported as a mild change^16^. In SR deficiency, the dopamine and serotonin pathways are usually severely affected^17^, and the low levels of aMT6s could be a reflection of the impaired brain serotonin status.

Patient 18, with a severe form of AADC deficiency (at age of 1 year, she showed hypotonia, oculogyric crises and dystonia), presented, as expected, an extremely low value of urinary aMT6s, which was related to the concomitant dramatic reduction of the CSF 5HIAA values.

It has been reported that L-dopa therapy may be toxic to serotoninergic neurons in cell cultures by oxidative mechanisms producing highly reactive quinone species that reduce serotoninergic neurons^18^. These findings also have been observed “*in vivo*” by similar oxidative mechanisms producing a significant decrease in serotonin and 5HIAA metabolite^19^, as well as affecting the behaviour and cognitive functions in animal models^19^. However, no differences were observed when compared urinary aMT6s values between naive GTPCH patients and those under L-dopa/carbidopa treatment. It is interesting that carbidopa treatment (an inhibitor of peripheral AADC activity) does not seem to affect urine aMT6s excretion, emphasizing that the contribution of peripheral melatonin is less relevant than that of pineal gland^1^.

Regarding patients under serotoninergic treatment, three AADC-deficient patients showed low aMT6s concentrations despite serotoninergic treatment. In patient 18, urinary aMT6s excretion increased after 3 months of MAOIs therapy, which suggests that this therapy improves serotonin and melatonin status, although the aMT6s value was still below the normal values. Two patients (patients 19–20) with a severe phenotype, extremely low CSF 5HIAA levels at diagnosis, and who were under MAOIs and PLP therapy, showed reduced aMT6s urinary excretion, while patient 21 with a moderate phenotype, who was also on therapy with MAOIs and PLP, showed normal urinary aMT6s values. An explanation for these data is that AADC is the most severe condition affecting brain serotonin status^20^ with a very reduced capacity of serotonin and melatonin biosynthesis. Further investigations are required to determine whether long-term MAOIs treatment can normalize aMT6s excretion in AADC patients. Patient 22 with a mild phenotype, who was on MAOIs, SSRI and melatonin therapy, showed higher aMT6s values than the RVs.

Regarding patients with BH_4_-related defects, patient 23 with SR deficiency was diagnosed at 23 months of age and presented the typical phenotype with psychomotor retardation, hypotonia, ataxia and extrapyramidal signs^16^. CSF neurotransmitter studies showed a classical SR deficiency pattern with severely decreased 5HIAA values in CSF. We found very low urinary aMT6s values despite the patient being under 5HTP therapy, which should have increased serotonin and melatonin biosynthesis (Fig. 1). His parents reported night urinary incontinency problems, a fact that could explain the unexpectedly low aMT6s values of the patient.

All DHPR-deficient patients had normal urine aMT6s values. Although these patients were under different treatment regimens, all of them received 5HTP, the serotonin precursor. Patient 24 was also on long-lasting treatment with SSRI, folinic acid and BH_4_. At 12 months of age (when she was already under this treatment), she presented normal CSF 5HIAA values. At 5 years of age, she showed high urine aMT6s values, which suggested that the combination of all these drugs may have led to this result. In fact, it has been suggested that some SSRI drugs may increase plasma melatonin values^21^. Patients 25 and 26 undertook only 5HTP therapy, and both showed normal aMT6s excretion, which supports the hypothesis that this serotonin precursor is effective in increasing urinary aMT6s excretion. However, the reduced size of our series strongly advises the development of clinical trials to confirm or rule out this hypothesis.

PTPS-deficient patient 27 was diagnosed by a newborn screening program, and at 1 year of age (when she was already on treatment), she showed mildly reduced CSF 5HIAA values. Currently, this patient is receiving 5HTP, BH_4_, and calcium folinate as serotoninergic drugs. The last CSF analysis reported normal CSF neurotransmitter concentrations, which suggest adequate therapy; the urinary aMT6s values were also normal. Unexpectedly, patient 28 had very low aMT6s values despite being under 5HTP and BH_4_ treatment, and had underwent a previous CSF 5HIAA analysis that showed normal results. In this case, the doctor and the family confirmed that the urine was correctly sampled; one explanation we have, may be related to individual variations on CYP1A2 activity. CYP1A2 is one of the major isoforms of cytochrome P450 in the liver and metabolizes a number of clinical drugs and several important endogenous compounds, such as melatonin^22^. There is a large inter-individual variability in the expression and activity of CYP1A2, which is caused mainly by genetic (several polymorphisms) and environmental factors^23^. Other explanation woud be a possible cerebral folate deficiency, since HIOMT needs S-adenosylmethionine (SAM) as a cofactor for melatonin biosynthesis. However, CSF 5-methyltetrahydrofolate values were normal in this patient.

A limitation of this study is that aMT6s determination strictly depends on a proper sample collection, which may be difficult in severely handicapped infants. Moreover, in some cases, the inter-individual variations of CYP1A2 activity^22,23^ could contribute to unexpected variations in urinary aMT6s excretion. Another important aspect of melatonin fluctuation is that it is greatly influenced by dim light, whereas very bright light can block melatonin production^11^. Finally, the simultaneous measurement of both CSF 5HIAA and urinary aMT6s in larger series of patients should be done to stablish a correlation between these two variables.

In conclusion, we studied 28 patients with different genetic serotoninergic metabolism defects and found that decreased excretion of aMT6s is frequently observed in all patients with more severe disorders. No consistent alterations were documented in the adGTPCH deficiency, which is the mildest disease studied here. Sulphatoxymelatonin can be a non-invasive good biomarker to estimate serotonin status in the brain, especially for treatment monitoring purposes.
